# Global Functional Connectivity Analysis Indicating Dysconnectivity of the Hate Circuit in Major Depressive Disorder

**DOI:** 10.3389/fnagi.2021.803080

**Published:** 2022-02-17

**Authors:** Pan Pan, Lu Wang, Chujun Wu, Kun Jin, Song Cao, Yan Qiu, Ziwei Teng, Sujuan Li, Tiannan Shao, Jing Huang, Haishan Wu, Hui Xiang, Jindong Chen, Feng Liu, Hui Tang, Wenbin Guo

**Affiliations:** ^1^National Clinical Research Center on Mental Disorders and Department of Psychiatry, The Second Xiangya Hospital of Central South University, Changsha, China; ^2^Department of Radiology, Tianjin Medical University General Hospital, Tianjin, China; ^3^Department of Psychiatry, The Third People’s Hospital of Foshan, Foshan, China

**Keywords:** major depressive disorder, fMRI, global-brain functional connectivity, network, hate circuit

## Abstract

**Background:**

Abnormalities of functional connectivity (FC) in certain brain regions are closely related to the pathophysiology of major depressive disorder (MDD). Findings are inconsistent with different presuppositions in regions of interest. Our research focused on voxel-wise brain-wide FC changes in patients with MDD in an unbiased manner.

**Method:**

We examined resting-state functional MRI in 23 patients with MDD and 26 healthy controls. Imaging data were analyzed by using global-brain FC (GFC) and used to explore the correlation of abnormal GFC values with clinical variables.

**Results:**

Increased GFC values in the left medial superior frontal gyrus (SFGmed) and decreased GFC values in the right supplementary motor area (SMA) were observed in the patients with MDD compared with the controls. The decreased GFC values in the right SMA had a positive correlation with vitamin D and Hamilton Anxiety Scale (HAM-A) scores.

**Conclusion:**

Abnormal GFC in the hate circuit, particularly increased GFC in the left SFGmed and decreased GFC in the right SMA, appears to be a new sight for comprehending the pathological alterations in MDD.

## Introduction

Typical signs of major depressive disorder (MDD) include persistent negative emotions, reduced volitional activity, and cognitive dysfunction. MDD brings a heavy burden to the patients and their families due to its significant characteristics of high morbidity and mortality. However, the pathophysiology of MDD remains unclear. Several abnormal biochemical serum indices exhibit potential diagnostic significance for MDD. For example, the levels of some monoamine metabolites are significantly associated with the incidence of MDD (Richelson, 2001; Moret and Briley, 2011). Vitamin D is involved in the synthesis of monoamine neurotransmitters as a common fat-soluble vitamin. Previous studies found a significantly negative correlation between vitamin D levels in serum and MDD occurrence in participants (Hoang et al., 2011; Jääskeläinen et al., 2015). In other words, vitamin D deficiency might be involved in the incidence of MDD. Patients with MDD have a higher rate of vitamin D deficiency than the general population (Anglin et al., 2013; Gelaye et al., 2015). Lower levels of vitamin D in serum reduced neurotransmitters such as cholinergic, dopamine, and norepinephrine, thereby increasing the risk of MDD. Lower levels of vitamin D in older adults may be considered a biomarker for the depressive state (Adibfar et al., 2016).

Several previous studies on the pathogenesis for MDD have focused on genetic factors, social environment, and other aspects. Nonetheless, no single hypothesis could perfectly explain all the characteristics and prognosis of this disease. The development of brain imaging has provided a new sight and method for exploring the pathogenesis of MDD in recent years. Researchers have achieved important progress in probing the neurobiological mechanisms of MDD from the aspects of brain structure and function. Neuroimaging studies indicated that MDD may be caused by disruptions within discrete brain networks rather than abnormalities in isolated brain regions (Wang et al., 2012). Neural networks that have been studied extensively in patients with MDD include the default mode network (DMN) and fronto-limbic network, where functional defects have been observed (Lee et al., 2008; Guo et al., 2015b; Scalabrini et al., 2020). Notably, the location of abnormally activated brain regions in previous studies focusing on brain dysfunction in MDD was involved in the main component of the hate circuit (Tao et al., 2013; Guo et al., 2015a). Hate, an intense basic human emotional response, plays an important role in psychological behavior and human evolution (Halperin, 2012). The hate circuit shows an altered activation when people watch stimuli they hate (Zeki and Romaya, 2008). Disgust at its core feeling of hate has been implicated in a wide range of psychological and mental conditions (Turnell et al., 2019). A number of studies have shown that hate is closely associated with depressive symptoms. For example, high levels of self-hate are directly related to the depressive state. Self-lathing in patients with MDD is associated with increased activity of the hate circuit (Surguladze et al., 2010; Turnell et al., 2019).

Resting-state functional connectivity (FC) between brain regions reflects its correlation with brain activity, which is an important tool to understand changes in FC between brain regions in patients with mood disorders. A number of studies have reported abnormal FC with inconsistent results in patients with MDD. For instance, patients with MDD in one study showed decreased FC between the medial orbitofrontal gyrus (Yu et al., 2021) and cerebellum (Zhu et al., 2020), whereas patients in another work exhibited increased homotopic FC (Liu et al., 2020) in the same brain regions.

One important factor contributing to these inconsistent results may be sample differences in age, population, locations, living style, etc. For example, patients with treatment-sensitive depression exhibited dysfunction in the frontoparietal top-down control network at specific frequency bands. However, patients with treatment-resistant depression showed abnormal FCs in the affective network, auditory network, visual network, and language processing cortex at the same frequency bands (He et al., 2016). Therefore, it is important to select first-episode, drug-naive patients with MDD to provide naive FC information to the pathophysiology of MDD.

Another important factor may be that previous studies focused on FC in specific brain regions. The region of interest (ROI) method was applied to these studies without a whole-brain examination. Critical areas associated with the core pathological alteration in MDD may be omitted by using this approach. Different ROI selections may result in different consequences. In this study, we adopted a data-driven approach, global-brain functional connectivity (GFC), to explore the pathophysiology of MDD and remedy the disadvantages of the ROI-based analysis (Preller et al., 2020). This method could be independent of the ROI selection. Therefore, GFC is helpful to obtain whole-brain FCs (Anticevic et al., 2014; Abdallah et al., 2017; Preller et al., 2018).

This study aims to utilize the GFC method, which belongs to the analysis method of the functional connectome, to probe the FC alterations in the whole brain of patients with MDD based on the aforementioned studies in MDD. We hypothesized that patients with MDD would exhibit GFC abnormalities in certain brain networks. Abnormal GFC in these brain regions may be related to the vitamin D levels in patients with MDD.

## Materials and Methods

### Participants

A total of 28 first-episode, drug-naive patients with MDD were involved from the Inpatient Department of the Second Xiangya Hospital of Central South University. The age of patients was between 16 and 45 years. Each patient was diagnosed and screened by two psychiatric experts according to the *Diagnostic and Statistical Manual of Mental Disorders*, Fifth Edition (DSM-5) (First, 2013). The participants were right-handed. The enrolled patients were first-episode patients and with the course of the disease not exceeding 1 year. All patients were required to complete the Hamilton Depression Rating Scale-17 (HAMD-17) (Hamilton, 1967), the Hamilton Anxiety Scale (Hamilton, 1959), and the Beck Depression Inventory-II (BDI-II) (Steer, 1993) to evaluate the clinical sign of MDD. Meanwhile, the cognitive functions of all patients were evaluated by the Repeatable Battery for the Assessment of Neuropsychological Status (RBANS) (Randolph et al., 1998). The exclusion criteria for the participants included serious physical disease and any other mental disorders consistent with DSM-5, any history of alcohol or drug abuse, any drug treatment to MDD, lipid-lowering therapy or vitamin D supplementation, pregnancy, and any contraindications to MRI scanning.

Thirty healthy controls who matched the patients with age, sex ratio, and education were involved by advertising in the local community. The DSM-5, Non-patients Version, was adopted to screen the healthy controls. The exclusion conditions included the history of serious neurological disorders, psychiatric disorders, and drugs or alcohol abuse in either the controls or their first-degree relatives.

All participants signed an informed consent form after obtaining an adequate explanation. Consent of the guardian was required for participants who aged under 18 years. This study is approved by the Ethics Committee of the Second Xiangya Hospital of Central South University and carried out in accordance with the Helsinki Declaration.

### Sample Collection

To avoid circadian rhythms interfering with the data, fasting blood samples for biochemical analyses were collected between 7 a.m. and 9 a.m. for all participants. Liver and kidney function, blood glucose, lipid series, and vitamin D levels were analyzed by serum tests.

### Neuroimaging Data Acquisition and Preprocessing

We used a Siemens 3.0 T scanner to obtain the resting-state images. The participants were required to lay on their back in a relaxed state and stay still during scanning. The neuroimaging data were automatically preprocessed using DPARBI and SPM8 in MATLAB (Yan et al., 2016). Detailed neuroimaging acquisition and preprocessing procedures are described in the Supplementary Material.

### Global-Brain FC Analysis

Global-brain functional connectivity is a measurement tool that computes the mean time series correlation between a given voxel with every other voxel for an unbiased method to determine the location of abnormal FCs (Anticevic et al., 2013, 2014; Murrough et al., 2016; Pan et al., 2019, 2021). The gray matter mask is produced by using the gray matter probability map (probability > 0.2) in the SPM8 software (Donishi et al., 2018). Such a threshold was chosen to eliminate the voxel with weak correlations that possibly originated from signal noises. A voxel-based GFC map was generated by composing GFC values of all voxels within the gray matter mask, where each voxel value represents the mean connectivity between the voxel and the rest of the brain. We computed the average Pearson’s coefficient (*r*) between the time series of each seed voxel and that of all other seed voxels throughout the whole brain after selecting each voxel within the gray matter mask as the seed voxel (Cole et al., 2010). GFC was calculated as average voxel-to-voxel connectivity throughout the gray matter mask as follows:


$$G\,F\,C\,a=\sum_{\text{b}=1}^{\text{n}}\frac{\text{r}\,\left({\text{T}}_{\text{a}},{\text{T}}_{\text{b}}\right)}{\text{n}-1}$$

where the Pearson correlation coefficient (*r*) of time series Ts for a pair of given voxels a and b was calculated. Finally, the mean correlation coefficient within the gray matter mask of the entire brain was calculated using the MATLAB software (Scheinost et al., 2016; Pan et al., 2021), and the data were normalized and converted into *z*-values using Fisher *r*-to-*z* transformation (Cole et al., 2010; Kaneoke et al., 2012; Thompson and Fransson, 2016).

The GFC maps between patients with MDD and controls were evaluated using two-sample *t*-tests. The framewise displacement (FD) values for each participant were calculated based on a previous study (Tozzi and Peters, 2017). The mean FD, education level, and age were substituted into the calculation as non-interest covariates. The threshold-free cluster enhancement (TFCE) was adopted to set the significance level as *p* < 0.05, a strict multiple comparison correction strategy that could achieve the optimal balance between the family-wise error rate and test-retest reliability/repeatability (Chen et al., 2018).

### Functional Connectivity Within the Hate Circuit Analysis

We performed a pairwise correlation analysis of six brain regions of the hate circuit in each subject by using the region-wise FC method in the RESTplus software package and obtained correlation coefficient *r*. Fisher *r*-to-*z* coefficients were adopted to transform into *z*-values to obtain a 6 × 6 FC matrix. The FC matrix between the patients with MDD and the controls was evaluated by two-sample *t*-tests.

### Statistical Analysis

Two-sample *t*-tests were conducted on continuous variables. A chi-square test was performed for gender distribution. Pearson correlation analyses were performed between GFC values in patients with MDD and clinical characteristics including vitamin D, blood lipid series, and HAMD-17, HAMA, BDI-II, or RBANS scores. The significance level was Bonferroni corrected at *p* < 0.05.

## Results

### Demographic and Clinical Characteristics

Five patients and four controls were excluded because they had excessive head movement during functional MRI (fMRI) scans after preprocessing the neuroimaging data. The final sample included 23 patients with MDD and 26 healthy controls. No significant differences in sex ratio, age, and years of education were observed between patients with MDD and healthy controls. The demographic and clinical characteristics of the participants are summarized in Table 1.

**TABLE 1:** Characteristics of the participants.

	Patients (*n* = 23)	Controls (*n* = 26)	*P*-value
Sex (male/female)	7/16	11/15	0.390^a^
Age (years)	23.17 ± 4.30	20.85 ± 3.13	0.118^b^
Education level (years)	14.13 ± 2.30	14.62 ± 2.06	0.213^b^
HAMD-17	29.13 ± 8.59		
HAMA-14	21.04 ± 6.02		
BDI-II	27.46 ± 10.35		
VITD-T	24.50 ± 8.89		
Blood glucose	3.69 ± 0.86		
TG	0.72 ± 0.31		
CHOL	3.75 ± 0.92		
HDL-C	1.61 ± 1.79		
LDL-C	2.08 ± 0.83		
Vocabulary learning	25.30 ± 5.49		
Story retelling	12.17 ± 5.25		
Immediate memory total score	37.48 ± 9.51		
Graphic copy	19.09 ± 1.35		
Line positioning	16.35 ± 1.85		
Visual span total score	35.43 ± 2.04		
Picture named	9.96 ± 0.21		
Verbal fluency test	18.35 ± 4.98		
Verbal function total score	28.30 ± 4.97		
Digit span	15.26 ± 1.21		
Coding test	51.04 ± 11.42		
Attention total score	66.30 ± 11.55		
Vocabulary memory	6.65 ± 1.97		
Vocabulary recognition	19.78 ± 0.52		
Story recall	6.70 ± 2.89		
Figure memory	15.61 ± 2.64		
Delayed memory score	48.74 ± 6.36		
Stroop word	98.30 ± 10.36		
Stroop Color	65.17 ± 13.33		
Stroop Color-word	39.04 ± 7.38		

*^a^A p-value was obtained using a chi-square test.*

*^b^The p-values were obtained using two-sample t-tests.*

*HAMD-17, Hamilton Depression Scale-17; HAMA, Hamilton Anxiety Scale; BDI-II, Baker Depression Inventory-II; VITD-T, vitamin D.*

### Group Differences in Global-Brain FC

**FIGURE 1:**
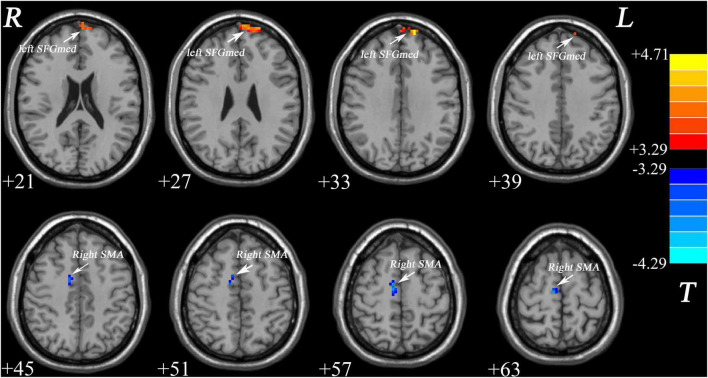
Increased GFC values in the left SFGmed and decreased GFC values in the right SMA were observed in patients with MDD relative to healthy controls. GFC, global-brain functional connectivity; SFGmed, medial superior frontal gyrus; SMA, supplementary motor area; MDD, major depressive disorder.

As shown in Figure 1 and Table 2, patients with MDD exhibited decreased GFC values in the right supplementary motor area (SMA) (*t* = −4.2841, *p* < 0.001) and increased GFC values in the left medial superior frontal gyrus (SFGmed) (*t* = 4.6535, *p* < 0.001) compared with the control group. No other differences were observed in the patients.

**TABLE 2:** Significant differences in GFC values between groups.

Cluster location	Peak (MNI)			Number of voxels	*T*-value
	x	y	z		
Patients > Controls					
Left SFGmed	−15	63	33	64	4.6536
Patients < Controls					
Right SMA	9	−15	63	42	−4.2841

*MDD, major depressive disorder; GFC, global functional connectivity; SFGmed: medial superior frontal gyrus; SMA: supplementary motor area; MNI, Montreal Neurological Institute.*

### Correlations Between Global-Brain FC and Clinical Characteristics

**FIGURE 2:**
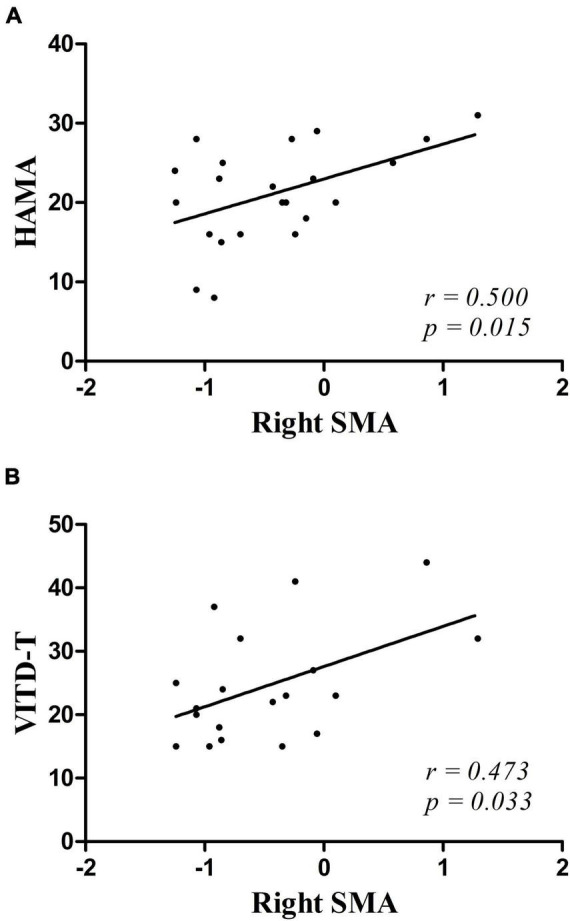
**(A,B)** Positive correlations were observed between the GFC values in the right SMA and the scores of HAMA and the vitamin D in patients with MDD. HAMA, Hamilton Anxiety Scale; VITD-T, vitamin D.

Decreased GFC values in the right SMA were positively correlated with HAMA scores (*r* = 0.500, *p* = 0.015) and vitamin D (*r* = 0.473, *p* = 0.033) (Figure 2). No significant correlations were found between the GFC values in the left SFGmed and the clinical variables. No relationships between GFC and age, years of education, blood lipid series, and HAMD-17, BDI-II, or RBANS scores were observed.

### Functional Connectivity Difference Within the Hate Circuit

**FIGURE 3:**
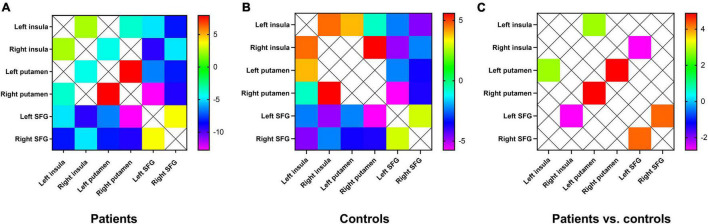
**(A)** Mean FC between the brain regions of the hate circuit in the patient group. One-sample t-tests on the FCs were conducted. The warm color denotes higher FC values, and the cold color denotes lower FC in the patients. **(B)** Mean FC between the brain regions of the hate circuit in the control group. The range from red to green denotes higher FC values. Blue and purple denote lower FC. One-sample t-tests on the FCs were conducted. **(C)** Increased FC between the left insula and the right insula, between the left putamen and the right putamen, between the left SFG and the right SFG and decreased FC values in the right insula and the left SFG were observed in patients with MDD relative to healthy controls. FC, functional connectivity; SFG, superior frontal gyrus; MDD, major depressive disorder.

Compared with controls, patients with MDD showed increased FC between the left insula and the left putamen, between the left putamen and the right putamen, and between the left SFG and the right SFG. FC between the right insula and the left SFG decreased (Figure 3).

## Discussion

Patients with MDD exhibit abnormal GFC in the brain regions of hate circuit in this study. Compared with the controls, significantly decreased GFC in the right SMA and increased GFC in the left SFGmed were observed in patients with MDD. The GFC in right SMA was positively correlated with vitamin D and the scores of HAMA.

Global-brain FC applies the basic image theory to study the overall connection between each voxel and every other voxel in the whole brain (Cole et al., 2010). This holistic approach was adopted to identify altered FC in the brain of patients with MDD and has proved to be very informative. This approach emphasizes qualitative data. Brain regions with abnormal FC may play a crucial role in coordinating massive brain activity patterns. Decreased GFC values may indicate low involvement of specific brain regions in diseases. Conversely, increased GFC values may indicate higher pathologies in specific brain regions (Buckner et al., 2009).

Superior frontal gyrus located in the upper prefrontal cortex (Shi et al., 2015) is considered to be composed of several subregions with different cellular structures (Petrides and Pandya, 1999, 2002), including Brodmann areas 6, 8, and 32 (Li et al., 2013). Previous studies on both task and resting-state fMRI have shown that different parts of SFG were involved in two anticorrelation networks, namely, task-positive network and DMN. These findings indicate that subregions exist in the SFG of humans (Li et al., 2013). Each subregion participates in different functional networks with its specific connectivity patterns of anatomical and functional levels. SFGmed plays as a pivotal node for DMN to participate in self-referential processing. The self-referential processing refers to the process of encoding, storing, and extracting information closely related to self-experience, which is often accompanied by special affective meaning and motivation (Northoff et al., 2006). Increased GFC in the left SFGmed indicates that it is abnormally activated in this brain region of patients with MDD at rest compared with controls in this study. Abnormal activation in SFGmed was closely related to excessive self-focus under the influence of MDD. The excessive self-focus often takes the form of rumination, which is a common clinical symptom in patients with MDD. Previous neuroimaging studies have revealed that patients with MDD of abnormal FC in SFGmed were more likely to cause excessive self-focus when they encounter negative emotional events (Lemogne et al., 2012). The suppression was enhanced abnormally in irrelevant information outside the self in patients with MDD when they were excessively self-focused. This reactive mode aggravated negative emotion and eventually formed into negative automatic thoughts (Morawetz et al., 2016). Previous studies have exhibited that a deceptive reaction requires more complex cognitive processing or stronger mind control, which may involve cognitive controls, including cognitive inhibition, conflict monitoring, resolution, and generativity (Spence et al., 2001; Nuñez et al., 2005). SFGmed is the physiological basis for performing cognitive control (Sohn et al., 2007; Ursu et al., 2009; Pourtois et al., 2010; Xu et al., 2021). SFGmed plays a role in working memory processing, and abnormal FC in this region is the basis of the deterioration of working memory for patients with MDD (Vasic et al., 2008). Significant activation in the SFGmed was associated with feigned long-term memory impairment, suggesting that SFGmed may underlie the physiological basis for the execution of feigned memory (Chen et al., 2015). The most active brain region frequently observed during lying is SFG, indicating that the region is the basic neural circuit for lying (Kozel et al., 2004; Spence et al., 2004). Increased GFC in SFGmed suggests that compensatory activation occurs in this brain region affected by MDD in this study. Patients with MDD showed increased cognitive control during feigned memory disorders.

Supplementary motor area belongs to the posterior SFG, located in the medial side of hemispheres, which is mainly activated by motor tasks (Li et al., 2013). Generally, SMA takes part in controlled movement as the secondary motor cortex. A previous study has found significantly increased FC in SMA in patients with MDD, suggesting that impaired function to this brain region may be involved in the pathophysiology of MDD (Frodl et al., 2010). This study reveals decreased GFC in the right SMA, which speculated spontaneous neural activity, and functional inhibition in severe depressive symptoms would cause thinking retardation, delayed speech, decreased initiative, and a series of involuntary motor behaviors such as akathisia and unconscious standing in several patients.

Tao et al. (2013) first reported FC alteration in the hate circuit of patients with MDD. They provided a perspective that the hate circuit may reflect reduced cognitive control over oneself and others in resentment generation. This study also focuses on FC within the hate circuit. The circuit includes the SFG, insula, and putamen. The SFG exhibited increased activities as well as enhanced activation in response to positive emotional stimuli. The putamen contains neurons in the preparation phase for motor behavior and participates in the perception of contempt and disgust. This brain region may have been involved in preparing offensive activity. The insula may be the physiological basis of activity in response to painful sensory stimuli. Damage to putamen and insula could affect the ability to recognize disgust signals in patients. The analysis of FC within the hate circuit exhibited abnormal FC between the SFG, putamen, and insula. Abnormal FC between these regions may be the physiological basis for the aggressive tendency and somatic discomfort in patients with MDD. Several studies have demonstrated that hate is closely associated with depressive symptoms (Overton et al., 2008; Surguladze et al., 2010). Reduced cognitive control in emotion is one of the important factors that cause MDD (Ebmeier et al., 2006). Patients with MDD have trouble in controlling negative thoughts. The hate circuit has been considered to be responsible for emotional and cognitive control regulation (Phillips et al., 2008; Fan et al., 2020). Impaired hate circuit might result in impaired cognitive control over pervasive internal self-loathing or hate of the external environment.

Decreased GFC values in the right SMA were positively correlated with vitamin D and HAMA scores. This finding indicates that decreased GFC in the right SMA may influence the anxiety level and provides theoretical foundation for the pathophysiology of anxiety signs in patients with MDD. Vitamin D deficiency has been shown to be causally associated with MDD (Milaneschi et al., 2014). Low vitamin D is closely related to the onset of MDD (Hoang et al., 2011), which probably explains that the treatment effect of MDD could be improved by adding vitamin D in addition to conventional antidepressant therapy. The cognitive function and spontaneous neural activity in patients with MDD may be affected by abnormal GFC in SMA due to alteration in vitamin D.

Several constraints should be taken into account in this research. First, the difference in age may have a bias effect on the present results although age has been treated as a covariate. Second, the vitamin D level may vary between genders, but no significant difference was found in this research. Finally, the alterations in gray/white matter were not explored. Therefore, the mechanism of change in gray/white matter underlying GFC remains unclear.

## Conclusion

Despite the limitation, this study presents that GFC abnormalities in the brain areas are associated with the hate circuit in patients with MDD. Abnormal GFC values in the right SMA might be one of the brain functional bases that cause alterations in the anxiety level. This study provides preliminary evidence that the vitamin D level in patients with MDD might be associated with abnormal FC in the hate circuit.

## Data Availability Statement

The original contributions presented in the study are included in the article/Supplementary Material, further inquiries can be directed to the corresponding authors.

## Ethics Statement

The studies involving human participants were reviewed and approved by the Ethics Committee of the Second Xiangya Hospital of Central South University. The patients/participants provided their written informed consent to participate in this study.

## Author Contributions

WG, JC, HT, and HW provided the conception of the work. LW, CW, SL, YQ, ZT, KJ, and SC collected the data. FL, PP, TS, HX, and JH were responsible for the data analysis and interpretation. PP drafted the manuscript. WG critically revised the manuscript. All authors contributed to the article and approved the final version of the manuscript.

## Conflict of Interest

The authors declare that the research was conducted in the absence of any commercial or financial relationships that could be construed as a potential conflict of interest.

## Publisher’s Note

All claims expressed in this article are solely those of the authors and do not necessarily represent those of their affiliated organizations, or those of the publisher, the editors and the reviewers. Any product that may be evaluated in this article, or claim that may be made by its manufacturer, is not guaranteed or endorsed by the publisher.
