# Supplementary material for: Global Functional Connectivity Analysis Indicating Dysconnectivity of the Hate Circuit in Major Depressive Disorder
Source: Front Aging Neurosci. 2022 Feb 17;13:803080. doi: 10.3389/fnagi.2021.803080 (PMC8891607; doi:10.3389/fnagi.2021.803080)
Supplement: Supplementary file 1 [file Data_Sheet_1.pdf]

Supplementary material

Title:

Global Functional Connectivity Analysis Indicating Dysconnectivity of the Hate Circuit in Major Depressive Disorder

Authors:

Pan Pan[a], Lu Wang[a], Chujun Wu[a], Kun Jin[a], Song Cao[a], Yan Qiu[a], Ziwei Teng[a], Sujun Li[a], Tiannan Shao[a], Jing Huang[a], Haishan Wu[a], Hui Xiang[a], Jindong Chen[a], Feng Liu[b], Hui Tang[a*], Wenbin Guo[a,c*]

Affiliation/address:

[a] National Clinical Research Center on Mental Disorders, and Department of Psychiatry, The Second Xiangya Hospital of Central South University, Changsha, Hunan 410011, China.

[b] Department of Radiology, Tianjin Medical University General Hospital, Tianjin 300000, China.

[c] Department of Psychiatry, The Third People's Hospital of Foshan, Foshan, Guangdong 528000, China.

# Corresponding authors:

Wenbin Guo

National Clinical Research Center for Mental Disorders, and Department of Psychiatry, The Second Xiangya Hospital of Central South University, Changsha 410011, Hunan, China

E-mail: guowenbin76@csu.edu.cn

Tel.: +86 731 85360921

Hui Tang

National Clinical Research Center for Mental Disorders, and Department of Psychiatry, The Second Xiangya Hospital of Central South University, Changsha 410011, Hunan, China

Email: tanghui2017@csu.edu.cn

Tel.: +86 7 31 85360921

Image acquisition and preprocessing

MRI images were obtained on a Siemens (Trio) 3T scanner at the Second Xiangya Hospital of Central South University. The participants were required to remain motionless and awake with their eyes closed. The participants used soft earplugs and foam pads to reduce the scanning noise and head motion. Resting-state functional images were obtained with a gradient-echo echo-planar imaging (EPI) sequence using the following parameters: repetition time/echo time = 2000 ms/30 ms, 30 slices, 64 × 64 matrix, 90° flip angle, 240 mm field of view, spatial resolution = 3.75 mm × 3.75 mm, 4 mm slice thickness, 0.4 mm gap, and 250 volumes lasting for 500 s. After the scan, each participant was asked some questions to confirm the wakefulness during the scan.

Functional images data were preprocessed by using the DPARBI software(Yan 2016). The fMRI time series were first corrected for within-scan acquisition time differences between slices and realigned to the first functional scan to correct for head motion. We excluded the participants whose head movement exceeding 2.0 mm of translation or 2° of rotation in any directions. All the realigned images were spatially normalized to the Montreal Neurological Institute in SPM8 and resampled to 3 × 3 × 3 mm^3 (Liu 2015) . After normalization, the images were smoothed (with an 8 mm full width at half maximum Gaussian kernel). The time series were further linearly detrended and temporally band-pass filtered (0.01–0.08 Hz). After that, several covariates were removed including Friston-24 head motion parameters acquired by rigid body correction, signal from a ventricular region of interest (ROI), and signal from a region centered in the white matter. The global signal was not removed as indicated

in a previous study(Hahamy A 2014).

Hahamy A, Calhoun V, Pearlson G, et al

2014 Save the global: global signal connectivity as a tool for studying clinical populations with functional magnetic resonance imaging. Brain Connect 4(6):395-403.

Liu, F., Guo, W., Fouche, J. P., Wang, Y., Wang, W., & Ding, J., et al

2015 Multivariate classification of social anxiety disorder using whole brain functional connectivity. Brain Structure & Function 220(1):101.

Yan, C. G., Wang, X. D., Zuo, X. N., & Zang, Y. F

2016 Dpabi: data processing & analysis for (resting-state) brain imaging. Neuroinformatics 14:339-351.
